# Variations in Leprosy Manifestations among HIV-Positive Patients, Manaus, Brazil

**DOI:** 10.3201/eid1504.081300

**Published:** 2009-04

**Authors:** Carolina Talhari, Christiane Matsuo, Anette Chrusciak-Talhari, Luis Carlos de Lima Ferreira, Marcelo Mira, Sinésio Talhari

**Affiliations:** Institute of Tropical Medicine of Amazonas, Manaus, Brazil (C. Talhari, C. Matsuo, A. Chrusciak-Talhari, L.C. de Lima Ferreira, S. Talhari); State University of Amazonas Faculty of Medicine, Manaus (C. Talhari, C. Matsuo, A. Chrusciak-Talhari, L.C. de Lima Ferreira, S. Talhari); Pontifical Catholic University of Parana, Curitiba, Brazil (M. Mira)

**Keywords:** leprosy, HIV, AIDS, letter

**To the Editor:** Contrary to early expectations, the co-occurrence of leprosy and HIV has not increased globally (*1*). However, most of the larger studies on the subject were conducted in the early to mid-1990s in African countries, and the research designs had limited power to describe the true effects of co-infection (*1*). Moreover, the introduction of highly active antiretroviral therapy (HAART), which has been used routinely in Brazil since 1996, altered the clinical evolution of HIV infection (*2*) and led to increasing reports of immune restoration inflammatory syndrome (IRIS) associated with leprosy (*3*,*4*). Although some researchers have argued that this association may not affect public health (*2*), its true importance remains to be clarified. Finally, leprosy has a wide range of clinical manifestations, which sometimes imposes a clinical challenge and may lead to misdiagnosis (*5*). Together, these factors may have helped mask the true scenario of leprosy and HIV co-infection, particularly in areas where these conditions are highly endemic. In this context, case reports from referral centers that reflect the broad clinical aspects of leprosy and HIV co-occurrence are important to increase clinicians’ awareness of both diseases.

We report 3 HIV-positive/AIDS patients who showed different clinical manifestations of leprosy; their conditions were diagnosed before and after HAART initiation. All patients lived in Manaus, the capital of the state of Amazonas in Brazil, an area where both leprosy and HIV infection are endemic. The 3 patients represent a sample from our 11-year experience with 21 patients with leprosy and HIV co-infection.

Patient 1 was a 29-year-old woman whose HIV-1 infection was diagnosed in May 2002 at antenatal examination. Her CD4 cell count in 2002 was 513 cells/µL. In November 2007, she sought treatment at the Institute of Tropical Medicine of Amazonas with a 3-month history of a single erythematous plaque on her left arm, which was clinically diagnosed as borderline tuberculoid (BT) leprosy. The patient’s sensitivity to pain was decreased. There was no nerve enlargement. Histopathologic examination confirmed the diagnosis, showing a granulomatous dermatitis with no acid-fast bacilli on Wade stain. At this time, her CD4 cell count was 342 cells/µL. HAART and multidrug therapy (MDT) for paucibacillary leprosy were initiated. The leprosy resolved, and the lesion disappeared within 2 months of therapy.

Patient 2 was a 22-year-old man who had neurocryptococcosis and HIV infection diagnosed in September 2007. At that time, he exhibited disseminated, infiltrated lesions on the trunk and upper and lower limbs. Borderline lepromatous (BL) leprosy was clinically diagnosed. Skin biopsy confirmed the diagnosis; the biopsy specimen showed a granulomatous dermatitis, foamy cells, and multiple acid-fast bacilli. His CD4 cell count was 6 cells/µL. HAART and MDT for multibacillary leprosy were prescribed. In February 2008, the patient was readmitted to the Institute of Tropical Medicine of Amazonas and died of nonspecified bacterial pneumonia and sepsis.

Patient 3 was a 23-year-old woman who had HIV-1 infection (CD4 cell count 435 cells/µL) diagnosed in November 2006 at antenatal examination. HAART was begun 3 months later. In August 2008, she sought treatment with a 3-month history of a single patch on the left leg with erythematous papules on its border (Figure). There was decreased pain sensitivity in the lesion and no nerve enlargement. At that time, her CD4 cell count was 372 cells/µL. Histopathologic examination showed tuberculoid granulomas consisting of lymphocytes and epithelioid cells. Wade staining showed no acid-fast bacilli. Histopathologic findings led to a diagnosis of BT leprosy. MDT for paucibacillary leprosy was promptly started, and HAART was continued. When she was last seen, in December 2008, the skin lesion had disappeared and she was still receiving MDT.

**Figure:**
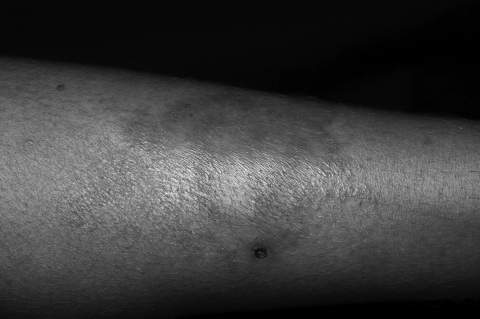
Skin lesion of patient 3, a solitary patch on the left leg with erythematous papules on the border.

The reliability of the cardinal signs of leprosy (hypopigmented or reddish patches with definite loss of sensation, thickened peripheral nerves, and positive skin smears or biopsy material) has been widely accepted (*5*). However, in some difficult cases, the definitive diagnosis relies solely on the histopathologic examination, which often depends on the experience of the pathologists working in referral centers. According to most pre-HAART studies, the clinical spectrum of leprosy seems to be preserved in HIV-positive and AIDS patients (*1*). This is in agreement with the course of disease in patient 1 (a typical BT lesion before initiating HAART) and patient 2 (a typical multibacillary leprosy in a full-blown AIDS background). For patient 3, a distinct outcome was observed: the appearance of an atypical BT lesion during HAART. Recently, we reported 3 cases of IRIS associated with leprosy in which BL leprosy shifted unexpectedly to BT leprosy (*4*,*6*). Host genetic make-up and unknown consequences of HIV-infection over specific leprosy immune mechanism may be implicated in these unusual outcomes. Further prospective studies should be performed to elucidate these findings.

Although previous studies have shown that HIV infection is not a risk factor for leprosy (*1*), clinicians should be aware of this potential co-infection, which may mimic different skin diseases. Moreover, reports of leprosy after HAART initiation have been described from countries where leprosy is not endemic (*7*). Precise diagnosis and prompt treatment of leprosy in co-infected persons are mandatory.
